# Diagnostic and Surgical Challenges in Parathyroid Neoplasia: An Extensive Analysis of a Single Endocrine Surgery Center Cohort of Patients

**DOI:** 10.3390/cancers17111783

**Published:** 2025-05-26

**Authors:** Razvan Simescu, Andra Piciu, Valentin Muntean, Alexandru Mester, Daniel Corneliu Leucuta, Doina Piciu

**Affiliations:** 1 Medlife-Humanitas Hospital Cluj-Napoca, 400664 Cluj-Napoca, Romania; 2 Department of Surgery, University of Medicine and Pharmacy “Iuliu Hatieganu”, 400012 Cluj-Napoca, Romania; 3 Department of Medical Oncology, University of Medicine and Pharmacy “Iuliu Hatieganu”, 400012 Cluj-Napoca, Romania; 4 Department of Oral Health, University of Medicine and Pharmacy “Iuliu Hatieganu”, 400012 Cluj-Napoca, Romania; 5 Department of Medical Informatics and Biostatistics, University of Medicine and Pharmacy “Iuliu Hatieganu”, 400349 Cluj-Napoca, Romania; 6 Doctoral School, University of Medicine and Pharmacy “Iuliu Hatieganu”, 400012 Cluj-Napoca, Romania; doina.piciu@umfcluj.ro; 7 Affidea Cluj-Napoca, 400487 Cluj-Napoca, Romania

**Keywords:** parathyroid tumors, pre- and intraoperative suspicion, bilateral neck exploration, *en bloc* resection, neuromonitoring

## Abstract

Background: Parathyroid neoplasia is a heterogeneous group of tumors, including parathyroid adenoma (PA), atypical parathyroid tumors (aPTs), and parathyroid carcinoma (PC). Differential diagnosis, especially preoperatively, between parathyroid carcinoma and the other two entities is challenging. The purposes of this study were to highlight the main differences between different parathyroid tumors and to evaluate how combined PC suspicion and intraoperative adjuncts can influence surgical decision-making and outcome-related issues. Methods: We performed a retrospective study of a database of patients diagnosed with parathyroid tumors who underwent surgical treatment at our endocrine surgery referral center between June 2019 and July 2024. Demographic, clinical, biochemical, imaging, intraoperative, immunohistochemical, and follow-up data were analyzed. Results: A total of 83 cases were included in our study, divided for analysis into PA (n = 67), aPT (n = 9) and PC (n = 7) subgroups. The clinical profile of the cohort showed a significant difference (*p* < 0.05) between the PA, aPT, and PC subgroups regarding the presence of palpable tumors (0% vs. 11.11% vs. 14.29%), both bone and kidney involvement (14.93% vs. 44.44% vs. 85.71%), and extensive disease beyond bone and kidney involvement (4.48% vs. 44.44% vs. 71.43%). PTH levels over five times the normal value were present at significantly different rates (*p* < 0.001), with higher rates in the aPT and PC subgroups (55.56% and 85.71%, respectively) compared with the PA subgroup (7.46%). Also, a significant difference (*p* < 0.001) was observed when analyzing extreme albumin-corrected serum calcium elevations over 14 mg/dL, with much higher rates in the PC subgroup (71.43%) compared to PA (1.49%) and aPT (33.33%). On preoperative ultrasonography, a significantly higher number of PCs presented diameters ≥ 3 cm (*p* < 0.001), depth-to-width ratios (D/W) ≥ 1 (*p* = 0.003), suspicious delineation (*p* < 0.001), and suspicious echotexture features (*p* < 0.001), compared to PAs. On preoperative US performed by the surgeon, suspicious features for thyroid cancer were identified in five more patients compared to the four identified by the initial US evaluation, and all (10.84% of all patients) were confirmed on final histopathology as papillary thyroid cancers. Intraoperatively, a significant difference (*p* < 0.001) regarding parathyroid macroscopic suspicious features, including adhesions to the thyroid gland, was seen between subgroups. When analyzing only cases with *en bloc* resection, we found that, in all PC cases, a combined preoperative suspicion was present, and in five cases an intraoperative suspicion was raised. Immunohistochemical data showed significantly different median Ki-67 indices between subgroups (1, 2, and 5; *p* = 0.008) and a different parafibromin staining profile between PC and aPT. Regarding intraoperative neuromonitoring use, a significantly lower incidence of voice changes related to the external branch of the superior laryngeal nerve was observed in the monitoring vs. non-monitoring group (57.14% vs. 12.5%, *p* = 0.019). Conclusions: Our findings confirm that, in a multimodal and combined diagnostic approach, early pre- and intraoperative PC suspicion can be raised in order to optimize surgical treatment and, thus, favorably influence the outcome. Utilizing all resources available, including intraoperative parathormone determination, laryngeal nerve neuromonitoring, and immunohistochemistry staining, can bring extra benefit to the management of these challenging cases.

## Simple Summary

This study compares different types of parathyroid tumors—benign, atypical, and malignant—highlighting the challenges in diagnosis and surgery. Malignant tumors tend to show more severe symptoms, elevated hormone and calcium levels, and distinct ultrasound features. The research underscores the importance of early suspicion and a combined approach using clinical, imaging, surgical, and pathology data to guide effective treatment.

## 1. Introduction

Parathyroid neoplasia is a heterogeneous group of tumors, including parathyroid adenoma (PA), atypical parathyroid tumors (aPTs), and parathyroid carcinoma (PC). Differential diagnosis between these entities is challenging. Compared to PA, which exhibits a female predominance, aPTs and PC are reported to be almost equally distributed between genders. Also, their peak incidence, in the fifth decade of life, is reported to be a decade earlier than that of PAs [1,2,3,4]. The etiology and pathogenetic mechanisms of both aPTs and PC are unknown. The majority of PC cases are sporadic, but cases have been shown also to be linked with genetic syndromes, such as hyperparathyroidism–jaw tumor syndrome (HPT-JT) or multiple endocrine neoplasia types A (MEN1) and 2A (MEN2A) [5,6]. HTP-JT syndrome has germline mutations of the Cell Division Cycle 73 (CDC73) gene, which is also mutated in up to 70% of sporadic PCs [7,8,9]. In contrast, somatic CDC73 mutations are very rare in aPTs [4].

The three pHPT entities present overlapping hypercalcemia-related signs and symptoms such as fatigue, weakness, muscle and joint pain, constipation, neuropsychiatric manifestations (depression, confusion), palpable neck mass, and renal (nephrolithiasis, nephrocalcinosis, kidney failure) and skeletal involvement (bone pain, osteoporosis, brown tumors). Still, PCs tend to have a higher frequency of symptomatic hypercalcemia and hypercalcemic crisis episodes. Also, an increased presence of a palpable neck mass and more system involvement, especially combined bone and kidney involvement, has been observed. Biochemical features that raise the suspicion of malignancy include serum calcium > 13–14 mg/dL and parathormone (PTH) 5–10 times above the normal upper limit; <10% of the tumors are non-secreting [10,11,12,13]. The clinical and biochemical profile of patients with aPTs seems to be similar but less severe than that of PC patients [4].

Ultrasonography (US) and technetium 99 m sestamibi scintigraphy (MIBI), with or without SPECT/CT, are the most used imaging studies to detect and localize the pathological parathyroids. A few studies have focused on the value of US in discriminating between malignant and non-malignant lesions, and several features have been identified [14,15,16,17,18]. CT and MRI can also be useful in detecting parathyroid neoplasia, including ectopic glands, invasion of the surrounding structures, or local and distant metastases [19]. In PC cases, ^18^F-FDG PET/CT (18-fluoro fluoro-deoxi-glucose positron emission tomography) and ^18^F-CH PET/CT (18-fluoro choline positron emission tomography) can aid in evaluating loco-regional and distant spread, potential residual disease, or recurrences [20,21,22].

The only definitive treatment for parathyroid neoplasia (non-malignant and malignant) is complete surgical removal of the pathological parathyroids, without capsular rupture. Bilateral parathyroid exploration (BPE) with four-gland dissection remains controversial, especially if preoperative dual imaging concordance paired with a significant intraoperative PTH (IOPTH) drop is seen [23,24,25,26]. For PC, *en bloc* resection with the ipsilateral thyroid lobe, including ipsilateral isthmus and any peritumoral tissue, is recommended [27,28,29], although several studies have shown that the extent of surgery for localized PCs did not influence disease-free and overall survival [30,31].

Associated prophylactic central neck dissection remains controversial [12]. Interestingly, *en bloc* resection can be a surgical option provided a high index of pre- or intraoperative suspicion is established, but PC is frequently a histopathologically surprising diagnosis [32]. On intraoperative exploration, PC suspicion can be raised based on several macroscopic findings that can also be present in aPTs, making the differentiation with PCs questionable [33]. Intraoperative adjuncts used in parathyroid surgery include IOPTH and intraoperative neuromonitoring (IONM) of the laryngeal nerves. While some authors consider IOPTH to be crucial in both malignant and non-malignant cases [10], others consider its value in PC to be limited [28]. Convincing evidence for the utility of IONM in parathyroid surgery is lacking [34], but a recent meta-analysis of 60 studies on the use of IONM in thyroid and parathyroid surgery demonstrated a statistically significant effect favoring its use in reducing the incidence of recurrent laryngeal nerve injuries [35].

On histology, PA is diagnosed if an encapsulated or demarcated parathyroid neoplasm with absent or markedly diminished stromal adipose tissue and with an adjacent peripheral rim of normal gland is found [36]. Historically, the microscopic features of PC were first defined by Schantz and Castleman in 1973, including fibrous trabeculae, nuclear atypia, mitotic figures, and capsular or vascular invasion within the tumor tissue [37]. The 2022 WHO classification of parathyroid tumors clearly states that PC diagnosis is restricted to parathyroid neoplasms that show angioinvasion, lymphatic invasion, perineural or intraneural invasion, local invasion into adjacent anatomical structures, or histologically/cytologically documented metastatic disease [38]. In the 2022 WHO nomenclature, “atypical parathyroid tumor” has replaced the term “atypical parathyroid adenoma”, reflecting that this is a neoplasm of uncertain malignant potential because it demonstrates cytological and architectural features similar to those of PC but lacks unequivocal invasive growth or distant metastases. Also, an emphasis was put on the judicious use of immunohistochemical (IHC) biomarkers such as parafibromin, E-cadherin, PGP 9.5, galectin-3, Ki-67 proliferation index, and many others to support the diagnosis of PC [37].

Postoperative outcomes can be influenced by transient or permanent complications such as vocal cord palsy, hypo- and hyperparathyroidism, and in the case of PCs, also by loco-regional recurrence of the disease or metastasis occurrence and disease progression. No specific guidelines for the surveillance of patients operated on for aPTs or PC exist to date.

Given that recurrences have been reported in about 50% of PC cases [39], even after a 20-year disease-free interval [40], a life-long monitoring of serum calcium and PTH (bi-annually for five years, and then yearly) and neck ultrasound (annually) has been advocated [12,13]. For aPTs, some authors suggest a close follow-up only in patients with large tumors and/or with complete loss of parafibromin expression [41,42], while others recommend annual follow-up for the first 5 years after surgery and every 2–3 years thereafter, regardless of parafibromin status [4].

The aim of our study was to highlight the main clinical, biochemical, imagistic, and pathological differences between PC, aPT, and PA cases, and to evaluate whether and how combined PC suspicion and intraoperative adjuncts influence surgical decision-making, outcomes, and follow-up.

## 2. Patients and Methods

### 2.1. Study Design and Setting

This study was a retrospective analysis of a prospectively collected database of patients diagnosed with parathyroid tumors who underwent surgical treatment at a single referral center (MedLife-Humanitas Endocrine Surgery Center Cluj-Napoca) between June 2019 and July 2024. The study was structured according to the Strengthening the Reporting of Observational Studies in Epidemiology (STROBE) checklist [7,43], ensuring a standardized approach to data collection, analysis, and reporting. Ethical approval was obtained from the institutional review board and from the Ethics Committee of the University of Medicine and Pharmacy “Iuliu Hatieganu” Cluj-Napoca, Approval No. AVZ.30/20.02.2024. All patients provided informed consent for data use at the time of their operations.

### 2.2. Patient Selection

This study included patients with histologically confirmed parathyroid tumors, classified as parathyroid adenoma (PA), atypical parathyroid tumor (aPT), or parathyroid carcinoma (PC), according to the WHO classification [38]. No cases of diffuse parathyroid hyperplasia were diagnosed histologically in our cohort. All cases underwent histopathological review, and only those with a definitive diagnosis of adenoma, atypical adenoma, or carcinoma were included. Eligible patients had complete preoperative clinical, biochemical, imaging, intraoperative, and follow-up data. Patients were excluded if they were diagnosed with parathyroid hyperplasia, secondary or tertiary hyperparathyroidism, if they had previous thyroid operations, if they had incomplete data recordings, or if they missed the follow-up controls.

### 2.3. Data Collection and Variables

Demographic, clinical, biochemical, imaging, intraoperative, and histopathological data were extracted from our institutional database. Demographic variables included age and sex. The clinical data retrieved were the presence of a palpable mass, symptoms (hypercalcemic crisis, muscle and joint pain, neurological symptoms, abdominal symptoms), and signs (bone and kidney involvement). Hypercalcemic crisis was defined as a serum calcium level exceeding 14 mg/dL, accompanied by clinical symptoms such as altered mental status, dehydration, or cardiac arrhythmias, necessitating emergency management.

The biochemical parameters analyzed were serum PTH, albumin-corrected calcium, 25-OH vitamin D levels, thyroid function parameters (FT4 and TSH), and peroxidase and thyroglobulin antibody levels. In our institution, the normal values for PTH were 15–65 pg/mL, using an electrochemiluminescence immunoassay (ECLIA) method. The localization studies were US neck ultrasound and MIBI scan with or without SPECT/CT in most patients, and CT or MRI in selected cases. Preoperative ultrasound was carried out by the same endocrine surgeon (R.S.) in all cases, and findings included tumor size, depth-to-width ratio (D/W), margin irregularity, inhomogeneous structure, calcifications, and cystic changes. Intraoperative features included tumor color and firmness, adhesions to surrounding structures, type of surgery, laryngeal nerves’ functional monitoring parameters, and IOPTH results. All cases of aPT and PC were reviewed based on the 2022 WHO criteria [38], and expression of IHC markers such as Ki-67, parafibromin, galectin-3, and E-cadherin was analyzed on formalin-fixed, paraffin-embedded tissue samples.

### 2.4. Surgery, Laryngeal Nerve Neuromonitoring, and Intraoperative PTH Assay

All patients underwent parathyroid exploration (bilateral in all cases except 3, in which video-assisted parathyroidectomy was associated with unilateral exploration) with parathyroidectomy performed by three experienced endocrine surgeons (R.S., V.M., and M.P.), following standardized surgical protocols. In cases with suspected malignancy based on preoperative and intraoperative findings, parathyroid and thyroid *en bloc* resection was performed, including the resection of adherent structures when necessary. In selected cases, IONM of the recurrent laryngeal nerves (RLNs) and the external branch of the superior laryngeal nerves (EBSLN) was used, particularly in patients undergoing concomitant thyroid surgery. The intermittent IONM (iIONM) was used in all surgeries where nerve monitoring was applied. The equipment used was the NIM-VITAL^TM^ Nerve Monitoring System (Medtronic).

For concomitant assessment of the 4 nerves (2 RLNs and 2 EBSLNs), neuromonitoring was performed through our own setup, using receiving needle electrodes inserted through the cricothyroid muscle and the cricothyroid membrane into the lateral arytenoid muscles. The rest of the IONM protocol was in accordance with the standards set by the International Neural Monitoring Study Group (INMSG) [44,45].

A surrogate intraoperative PTH assay (sIOPTH) based on the Miami criteria [46] was carried out in the majority of the cases, with blood samples being drawn during the operation from the internal jugular vein. The term “surrogate” is used here because we did not have the necessary equipment to perform true IOPTH assays and, thus, the results of the serum PTH levels were obtained from the laboratory only after the operation ended.

### 2.5. Outcome and Follow-Up Measures

All patients included in this study had a postoperative evaluation approximately 6 weeks after surgery. All patients with PC and those with postoperative complications had regular check-ups. Follow-up data included biochemical parameters (serum parathyroid hormone, corrected calcium, and 25-OH vitamin D levels), along with FT4 and TSH levels in case of concomitant thyroid operation. Vocal cord function was investigated by laryngoscopy. The function of the EBSLN was indirectly assessed by inquiring for specific voice changes experienced by the patient, such as lower-pitch sounds, weak voice projection, voice fatigue, and the inability to sustain an “S” and “Z” pronunciation over 5 s [45].

### 2.6. Statistical Analyses

Counts and percentages were used to describe categorical data. Medians and interquartile ranges were used to describe quantitative data that did not follow the normal distribution. Comparisons between two or three groups concerning categorical data were performed using the chi-squared test or Fisher’s exact test (in case of low expected frequencies). Comparisons between two groups regarding quantitative data not following the normal distribution were performed with the Wilcoxon rank-sum test, while comparisons between three groups were performed with the Kruskal–Wallis test (non-parametric post hoc tests were used afterwards). For all statistical tests, a 0.05 level of significance was used, and the two-tailed *p*-value was computed. All analyses were performed in the R environment for statistical computing and graphics (R Foundation for Statistical Computing, Vienna, Austria), version 4.3.2 [47].

## 3. Results

### 3.1. Epidemiological and Clinical Characteristics of the Patients

There were 83 patients included in our study, divided into a PA subgroup (n = 67, 80.72%), aPT subgroup (n = 9, 10.87%), and PC subgroup (n = 7, 8.43%). The characteristics of the study cohort are presented in Table 1. The median age at diagnosis did not significantly differ between the three subgroups. A female predominance was present in the whole study population, but in the PC subgroup the female/male ratio was almost 1. The clinical profile of the cohort showed a significant difference (*p* < 0.05) between the PA, aPT, and PC subgroups regarding the presence of palpable tumors (0% vs. 11.11% vs. 14.29%), combined bone and kidney involvement, and marked (>2) systemic involvement (4.48% vs. 44.44% vs. 71.43%). Two patients, one from the aPT subgroup and the other from the PC subgroup, experienced a hypercalcemic crisis. Among the patients with parathyroid cancer who had multiorgan involvement, the most frequently affected systems, in descending order, were (a) the skeletal system (osteitis fibrosa cystica, bone pain, or fractures), (b) the renal system (nephrolithiasis or nephrocalcinosis), (c) the gastrointestinal system (nausea, constipation, or pancreatitis), and (d) neuropsychiatric symptoms (fatigue, depression, confusion).

No significant family history or personal irradiation history was recorded. Concomitant thyroid disease was present in about half of the cases of all three subgroups, without significant differences in distribution. In our cohort, no clinical or genetic data suggested MEN1-, MEN2-, or CDC73-related syndromes in the carcinoma cases, but genetic testing was not routinely performed, which is a limitation of our study.

### 3.2. Preoperative Biochemical Characteristics of the Patients

Biochemical characteristics are also presented in Table 1. Non-functioning tumors were not identified in our cohort of patients. Significant differences in median PTH (*p* < 0.001) and albumin-corrected serum calcium (*p* = 0.006) were observed between the three subgroups of patients. PTH levels’ extreme elevations (PTH ≥ 5 times the normal value; in this study, “normal value” refers to the upper limit of the reference range) were present and significantly different, with higher rates in both the aPT and PC subgroups (55.56% and 85.71%) compared with the PA subgroup (7.46%). Similar significant differences (*p* < 0.001) between the three subgroups were observed when analyzing extreme elevations (Ca > 14 mg/dL) of albumin-corrected serum calcium levels, with much higher rates in the PC subgroup compared to PA and aPT. No significant difference (*p* = 0.975) in median 25-OH vitamin D levels (*p* = 0.975) and hypovitaminosis D rates was observed between PA, APT, and PC. The presence of concomitant Hashimoto’s thyroiditis was not significant different (*p* = 0.587) between the subgroups.

### 3.3. Preoperative Imaging Results

Only three (3.61%) patients were operated on without preoperative localization of their parathyroid glands. Preoperative ultrasound was able to localize pathological parathyroid glands in 72 (86.75%) cases. All malignant parathyroids were identified and evaluated by US. A MIBI scan with or without SPECT/CT was performed to localize the parathyroids in 61 (73.49%) cases, with accurate localization in 95.08% of them. No ectopic parathyroids were identified. In six cases, isotope imaging was the only method to identify and localize pathological glands. CT or MRI was carried out in 13 (15.66%) cases, and in 2 of them they were the only methods to identify the parathyroids.

Pathological parathyroid US features are summarized in Table 2. On US scans, significant differences in the maximum median parathyroid diameter (*p* < 0.001) were seen between PA and APT, and between PA and PC. The median D/W ratios were seen to be significantly different (*p* = 0.005) between PC and both non-malignant subgroups. The analysis of the US findings also revealed that, compared to PAs, a significantly greater number of PCs presented diameters ≥ 3 cm (*p* < 0.001), D/W ratios ≥ 1 (*p* = 0.003), suspicious delineation (*p* < 0.001), and suspicious echotexture features (*p* < 0.001). For aPTs, the results were intermediate between those of PA and PC regarding D/W, median diameter, suspicious delineation, and echotexture features.

### 3.4. Intraoperative Data

Intraoperative suspicion of parathyroid malignancy was primarily based on the operating surgeon′s evaluation, considering macroscopic features such as tumor size, adherence to adjacent structures, fibrous bands, and invasion. Four endocrine surgeons participated in the study. To ensure consistency, the surgeons adhered to a standardized intraoperative checklist and held preoperative tumor board meetings to align on diagnostic and operative expectations.

Most of the cases (96.39%) received cervicotomy, and a minority of only PA patients (4.48%) received MIVAP. In all cervicotomy cases, a BPE of the four parathyroid glands was performed, and in four PA cases (5.97%), a double adenoma was identified and resected. The three patients with MIVAP received concordant US and MIBI localization studies; thus, only a unilateral parathyroid exploration (UNE) was performed. Table 3 shows the details of the intraoperative data.

A significant difference (*p* < 0.001) regarding parathyroid adherences to the thyroid capsule was observed between PC and PA, and between PC and aPT. No statistical differences between subgroups were observed regarding thyroid capsule adherences to the surrounding tissues, and especially to the strap muscles (as seen in Hashimoto’s thyroiditis cases). Also, a significant difference (*p* < 0.001) was identified in the analysis of other parathyroid macroscopic suspicious features (grayish-white, lobulated, firm), which were present in all but one PC and in almost half (44.44%) of the aPTs.

All cervicotomy cases had planned bilateral exploration of all four parathyroid glands, and four (4.82%) double PAs were identified intraoperatively. Of the PA, aPT, and PC subgroups, 65.67, 55.56, and 14.29%, respectively (*p* = 0.033), underwent only parathyroid excision. With significant differences between the three subgroups, the corresponding percentages for *en bloc* resection were 14.93, 22.22, and 85.71 (*p* < 0.001).

Also, concomitant thyroid surgery was significantly more common in PC compared to the PA and aPT subgroups (*p* = 0.018). None of the PC patients had a concomitant prophylactic or curative central compartment lymph node excision. Surrogate intraoperative parathormone assay (sIOPTH) was carried out in 84.34% of the patients.

In all but one patient (with <50% drop but within normal range), a significant reduction according to the Miami criteria (>50% drop at 10 min) was observed. Intraoperative neuromonitoring (IONM) was used in 34 (40.96%) patients, without significant differences between subgroups.

When analyzing only cases with *en bloc* resection, we found that in all PC cases a combined clinical, biochemical, and US suspicion was present, and in five cases an intraoperative suspicion was raised (Table 4). Regarding aPT, only one of the two cases with *en bloc* resection had strong preoperative PC suspicion features, but not intraoperative ones. In the 10 PA cases, suspicious features were rare, and all had concomitant thyroid pathology (nodular goiter, PTC suspicion, and Hashimoto’s thyroiditis) with indication for thyroid resection.

### 3.5. Immunohistochemistry Findings

Immunohistochemical data are also presented in Table 5. Both the median Ki-67 indices and rates over 5% were significantly higher in the PC subgroup compared with aPT and PA. Parafibromin, galectin-3, and E-cadherin stains were performed only on aPT and PC samples. Parafibromin staining was negative in 28.57% of the PCs and 11.11% of the aPTs, while it was positive in 71.43% and 88.89%, respectively (*p* = 0.035). No clear differences were observed between the aPT and PC subgroups regarding the rates of positive galectin-3 (*p* = 0.629) and negative E-cadherin (0.175) stains.

### 3.6. Concomitant Thyroid Pathology

On initial referral for neck US, a total of 42 (50.6%) patients were diagnosed with nodular thyroid disease, with concomitant thyroid cancer suspected in four cases. On preoperative US performed by the surgeon, suspicious features for thyroid cancer were identified in five more patients. Thus, the total number of patients with preoperative thyroid cancer suspicion was nine (21.43%): seven (10.61%) with PA and two (28.57%) with PC (Table 6). All nine tumors (10.84% of all patients in our cohort) were confirmed on final histopathology as papillary thyroid cancer (PTC). Microcarcinomas (PTMCs) were diagnosed in three (33.33%) patients, and double PTCs were diagnosed in four (44.44%) cases. Most of the cases exhibited modestly elevated serum PTH levels (77.78%) and normocalcemic or mildly elevated albumin-corrected serum calcium (88.89%).

### 3.7. Outcome and Follow-Up

Complications registered at the 45-day postoperative check-up are presented in Table 7. No significant overall distribution difference (*p* = 0.128) between the three subgroups was seen regarding PTH levels, although above-normal values were present in almost half (three out of seven) of the PC patients. Serum calcium levels were normal in all patients with elevated PTH, and they were also within the normal range in all patients with normal or low PTH levels, with no differences between the groups; the median (IQR) albumin-corrected serum calcium (mg/dL) was 9.02 (8.6–9.8). Also, low vitamin D levels were identified in about half of all patients, without statistically significant differences between subgroups (*p* = 0.922). Vocal cord palsy, identified by direct or indirect laryngoscopy, was present in seven cases (6%), without significant distribution between the PA, aPT, and PC cases. EBSLN-related voice changes were identified in 13 (15.66%) of the patients, with significant overall distribution differences (*p* = 0.024) between subgroups.

Subsequent follow-ups (every 6 months) were carried out in all PC cases, in most of the aPT patients (seven out of nine), and in PA patients with hypoparathyroidism. The mean follow-up time for PC patients was 18 ± 4.9 months (range 14–46), and for aPT patients it was 19.9 ± 10.4 months (range 16–65). There was no significant difference between the aPT and PC subgroups regarding the persistence of disease, and no recurrences were recorded. Two PC patients had persistent disease at 14 and 16 months after surgery, with the first being also diagnosed with pulmonary metastasis.

Of the aPT subgroup, one patient had persistent disease at 6 months after surgery, which was still present at the last check-up 65 months after the operation, but without imagistic evidence of disease. Of the two patients with low PTH at the first follow-up (both belonging to the PA subgroup), only one was diagnosed with persistent hypoparathyroidism at 6 months after surgery. Patients with vocal cord palsy or EBSLN-related voice changes were also checked at 6 months after surgery for permanent changes, and the results are presented in the next subsection.

#### Laryngeal Nerve Neuromonitoring and Related Outcomes

The outcomes of vocal function in patients who were operated on, with and without laryngeal nerve neuromonitoring assistance, are presented in Table 8. Transient and permanent vocal cord palsy was present in 4.82% and in 1.20% of the patients, respectively. In our study, all patients who reported voice changes suggestive of EBSLN injury underwent laryngoscopic examination postoperatively to assess their vocal cord mobility. No cases of vocal cord paresis or paralysis were observed, consistent with EBSLN involvement rather than recurrent laryngeal nerve injury. The vocal cord palsy rates were comparable between the monitored and non-monitored groups (5.88% vs. 6.12%, *p* = 1). A non-significant trend toward fewer voice changes related to the EBSLN was observed in the IONM group (8.82% vs. 20.41%, *p* = 0.153). When analyzing only cases with concomitant thyroid surgery, neuromonitoring was associated with a significant lower incidence of EBSLN-related voice changes (12.5% vs. 57.14%, *p* = 0.019). Vocal cord palsy remained non-statistically different between groups (6.25% vs. 14.29%, *p* = 0.586).

## 4. Discussion

Parathyroid carcinoma (PC) is a very rare disease, representing 0.005% of all malignancies, accounting for 0.5–5% of all primary hyperparathyroidism (pHPT) cases, and with an increasing incidence, estimated at 3.5–5.7 cases per 10 million people [2,7,13,14,48]. In most series of patients, the incidence of aPTs is also low, ranging between 0.5 and 4.4% of all pHPT cases, but up to 14% in Asian reports [4]. The present literature lacks studies with detailed and combined comparisons of clinical, biochemical, imaging, histological, and prognostic features between PA, aPT, and PC. To deepen the common knowledge of the rare forms of pHPT—namely, aPT and PC—and to evaluate how pre- and intraoperative PC suspicion and intraoperative adjuncts can influence surgical decision-making and outcomes, we retrospectively analyzed our prospectively maintained database of operated parathyroid cases.

In our study, almost half of the patients in the PC subgroup were male, in contrast to the distribution in both non-malignant subgroups, where females were clearly predominant, which is in line with previous data [2,3,38]. The median age at diagnosis did not differ between the subgroups, which is in line with a previous Finnish nationwide study of the three pHPT entities [3]. The median age of our PC patients was 57 years, similar to some reports [49], but different from other studies, where a tendency towards younger age was observed in PC compared to non-malignant cases [50,51]. This difference might be explained by the small number of PC cases and the fact that no familial cases, which are younger at diagnosis [4], were identified in our cohort of patients.

The clinical profile of our cohort showed significant differences between PC and PA cases regarding patients with suspicious features such as palpable tumors, combined bone–kidney involvement, and over two systems affected by the disease, which is in line with previous findings [52,53,54,55]. Regarding aPTs, we found intermediate percentages between the other two subgroups. In 2019, a review of data from case series with comparative analyses and case reports, including 672 patients, concluded that atypical parathyroid adenoma patients have a clinical profile more severe than that of PA patients, and similar to but not as severe as that seen in PC patients [4].

In our cohort, the median preoperative serum PTH levels differed significantly between PA, aPT, and PC, with the highest concentrations in PC patients. The median albumin-corrected serum calcium levels differed only between PC and the other two subgroups. Interestingly, insufficient vitamin D levels were present in more than half of the investigated patients (56.62% of the entire cohort), but this was not statistically different between the subgroups. Serum calcium > 14 mg/dL and PTH 5–10 times above normal, features previously shown to raise suspicion of malignancy [10,11,12,13], were distributed significantly (*p* <0.001) in favor of PC vs. PA and in the aPT vs. PA subgroups. Regarding aPT patients, their biochemical profile was more similar to that of PC patients, a feature that was also seen in the largest review and meta-analysis conducted by Cetani et al. in 2019 [4], and also in a large monocentric cohort study by Saponaro et al. in 2021 [56]. The fact that both the clinical and biochemical profiles of aPT patients were more in line with those of PC patients led us to speculate that the decision on the extent of surgery (PE only vs. *en bloc* resection) should not be made based solely on such elements.

To date, the most utilized and optimally regarded methods for the localization of pathological parathyroids in pHPT are neck US combined with MIBI [57,58,59]. A meta-analysis on US’s performance showed a pooled detection rate of 76.1%, with a reported range between 48.3% and 96.2% [60]. Also, studies on surgeon-performed US from high-volume centers have reported sensitivities of up to 87% for localizing pathological parathyroid glands to the correct quadrant of the neck [61]. In our cohort, only three patients were operated upon without preoperative pathological parathyroid localization. All referred patients were reexamined by one of the two operating surgeons (R.S.) proficient in neck US, and preoperative US localization of pathological parathyroids was achieved in 86.75% of them (72 patients). In the remaining 11 patients, localization was carried out by MIBI, CT, or MRI scans, supporting the results of Christakis et al. [62], who found that the sensitivity of every single preoperative procedure was about 80%, and up to 95% or more when the three methods were used in conjunction.

A few case–control studies identified several US features as being significantly different in PC vs. benign parathyroid tumors (PA and parathyroid hyperplasia): maximum diameter [14,15,16,17], irregular shape [14,15,16,17], thick capsule [14], uncircumcised margins [15,17], infiltration in the surrounding structures [15,16,17], heterogeneous echotexture [14,15,16,17], the presence of calcifications [15,16,17], cystic changes [17], and D/W ratio [14,17]. These studies, along with a large study on 286 patients with PC [63], also showed that the mean or median diameter of the PC tumors was around 3 cm. In our study, and to simplify the analysis, we merged all of the delineation features and all of the echotexture features of the tumors into two separate categories. However, similar to previous findings, our results showed significant differences between PA and PC tumors in all of the aforementioned features. There have been no studies on the comparison of US characteristics between aPT and PA or PC, and our data show that this type of differentiation is questionable.

The adequate surgical procedure depends on the pre- and intraoperative suspicion of PC, and this might also affect local recurrence and mortality rates. PC suspicion can be raised if, upon intraoperative exploration, an enlarged parathyroid with greyish-white coloration, of firm or hard consistency and dense fibrous capsule, also adherent to the surrounding tissues, is found [32]. These features can also be present in aPTs, leading to potential overtreatment of aPTs and undertreatment of PC cases if the operation is based solely on intraoperative suspicion [21,32].

In our PC cases, there was a clear link between combined pre- and intraoperative suspicion and the surgical procedure performed (Table 4), which was *en bloc* resection in six out of seven cases (85.71%). This is in concordance with the results of a recent study published in 2023 by Kawai et al., who analyzed the impact of pre- and intraoperative cancer suspicion on the choice of surgical procedure [64]. Still, we also performed *en bloc* resection for aPT (2 cases) and PA (10 cases).

Especially for most PA cases, no preoperative suspicion of PC existed, and intraoperative suspicion might be explained by the fact that 4 out of the 10 cases had concomitant Hashimoto’s thyroiditis with adhesions between the thyroid capsule and the surrounding tissues. The remaining six cases had concomitant thyroid pathology with surgical indications, and we speculate that the *en bloc* resection was just the preference of the surgeon to resect both glands together. Also, in our opinion, when evaluating adhesions of the parathyroid gland to the surrounding tissues, especially to the thyroid gland, the surgeon must be aware of concomitant thyroid pathology that can influence their presence.

The utility of IOPTH is still controversial among endocrine surgeons, with some considering BPE to be the best approach in all cases [22], while others state that IOPTH is unnecessary in cases of preoperative imaging concordant with single-gland disease [65,66,67,68]. Current guidelines recommend the use of IOPTH only in minimally invasive parathyroid surgery when preoperative localization with MIBI scan and US is not concordant or negative, or in case of re-interventions [56,68,69,70]. We performed BPE and UPE in 96.39% and 3.61% of the cases, respectively. The surrogate IOPTH used in 84.33% of the entire cohort confirmed the successful removal of the hypersecreting parathyroids (including four double adenomas) in all but one case. This confirms that when ectopic disease is preoperatively excluded by MIBI scan, BPE is still an efficient method to ensure operation success, especially in hospitals were IOPTH technology remains prohibited due to financial reasons.

Currently, it is generally believed that a Ki-67 cut-off level of 5% can help distinguish benign parathyroid tumors from PCs, but the 2022 WHO classification does not consider the proliferation index as a diagnostic criterion for PC [37]. Major studies on Ki-67 activity in parathyroid tumors reported that a Ki-67 index > 5% in PC, aPT, and PA ranged between 0% and 85.7%, 67%, and 57.7%, respectively [71].

In our cohort of patients, none of the aPTs had a proliferation index > 5%, and there was a significant difference between PA and PC in favor of PC patients. Also, the median Ki-67 differed significantly between the subgroups, with higher percentages in PC, but with overlapping ranges, similar to a previous nationwide study on parathyroid neoplasms in Finland by Ryhänen et al. [3]. Still, in another study by Quinn et al., no significant differences between cohorts of aPT and PC patients were found [72]. In previous studies, a high Ki-67 index was associated with PC recurrences [73,74,75,76]. We could not evaluate this aspect, as our PC patients did not experience any recurrences to the time of their last follow-up visit, which was relatively short.

Inactivated mutation of the CDC73 gene results in the loss of parafibromin (a tumor-suppressor protein) function and immunohistochemical expression. Loss of parafibromin stain has been linked with the diagnosis and poor outcomes in PC [77,78], as well as metachronous diseases in other glands or malignant behavior of aPTs [79,80,81]. In their 2022 review, Uljanovs et al. reported that, in the main studies of parafibromin expression in parathyroid tumors, loss of parafibromin was present in 0–17.6% of PAs, in 0–54.3% of APTs, and in 20–100% of PCs [71]. In our study, we found significant differences (*p* = 0.035) in parafibromin staining between the PC and aPT subgroups. A completely negative stain was observed in two (28.57%) PCs and in one (11.11%) aPT. Interestingly, a weak stain was observed in 42.86% of the PCs and in none of the aPTs, but this could have been due to the staining interpretation of the pathologist.

In their review of the literature, Davis et al. found five studies that bring evidence of galectin-3 overexpression as being indicative for PC diagnosis [82], with positive stains in between 40% and 92.3% [83,84,85,86,87]. In contrast, in one report by Mohammed et al., only 15% of PCs showed positivity, with 6% sensitivity and 29% specificity [88]. In two of the studies, 47.4% and 71.4% of aPTs were found to be galectin-3-positive [85,86]. Also, in their systematic review on atypical parathyroid adenoma articles, Cetani et al. found that galectin-3 overexpression ranged between 32% and 100%, a rate similar to that of PCs [4]. In our analysis, galectin-3 overexpression was not significantly different (*p* = 0.629) between the PC and aPT subgroups, and the percentages (28.57% and 11.11%, respectively) were below the ranges reported in the literature.

Several studies have explored the role of E-cadherin expression in parathyroid tumors [73,75,89]. In 2009, Fendrich et al. were the first who showed significant differences in the loss of its membranous expression in PCs (all nine patients) vs. PAs [89]. This was confirmed in 2024 by a larger study by Hu et al., who compared PCs (47%) with combined PAs and aPTs (18.6%) [75]. In contrast, the results of Silva-Figueroa et al.’s study from 2019 showed no statistical differences between the PC (61%), aPT (50%), and PA (40%) subgroups, but by combining the loss of E-cadherin expression with other biomarkers, such as Ki-67, parafibromin, galectin-3, and PGP9.5, the differential diagnosis of PC with non-malignant parathyroid tumors could be supported [73]. In our study, there was no statistically significant difference between the PC and aPT subgroups, but only two (28.57%) of the PCs and none of the aPTs had loss of E-cadherin expression.

Previous studies have reported that approximately 50% of patients operated upon for pHPT had concomitant thyroid nodular disease [90,91]. In our study, concomitant thyroid nodules were present in more than half of the entire cohort of patients, with no significant distribution differences between the three subgroups. On preoperative US performed by the surgeon, suspicious features for thyroid cancer were identified in nine patients, i.e., five more cases than the initial US evaluation. All nine tumors (10.84% of all patients in our cohort) were confirmed upon final histopathology as papillary thyroid cancers (PTCs). This highlights the utility of preoperative concomitant US evaluation of the thyroid, which may change surgical management to eliminate possible thyroid surgery in the future, as demonstrated by others [92,93,94,95,96]. A systematic review and meta-analysis in 2024 showed that, in operated pHPT patients, the pooled prevalence rate of papillary thyroid cancer was 8% [97]. The slightly higher prevalence of PTC in our cohort might be explained by the fact that we excluded patients with parathyroid hyperplasia from our analysis.

Several studies have explored the potential role of elevated PTH and calcium levels in thyroid malignancy, but the results are contradictory. While one study on 59 symptomatic pHPT patients showed that higher concentrations of PTH were associated with a higher risk of differentiated thyroid cancer [98], two other studies showed no correlation [99,100]. In contrast, two studies, each on over 300 patients, showed significantly lower serum PTH levels in pHPT patients with concomitant PTC compared to patients with benign thyroid nodules [101,102]. Also, the study of Xue et al. found a significant negative correlation between the albumin-corrected serum calcium level and the presence of thyroid cancer in patients with pHPT [99]. In our cohort, most of the patients with pHPT and PTC had moderately elevated PTH (77.78%) and normocalcemic or mildly elevated albumin-corrected serum calcium levels (88.89%), but interestingly, when pHPT due to PC was associated with PTC (two cases), higher levels of PTH (in both cases) and calcium (one case) were recorded.

No specific guidelines exist to date for the post-surgical surveillance of both aPT and PC. For PC, most authors and centers, based on their own experience, recommend a lifetime follow-up for all patients [26]. For aPT, some authors recommend a closer follow-up of patients with tumors showing negative parafibromin staining [3,40,41,77], while others perform annual evaluations for the first 5 years, and every 2–3 years thereafter [4]. At the first follow-up (approximately 45 days after surgery), no significant overall distribution difference (*p* = 0.128) between our three subgroups was seen regarding PTH levels, although above-normal values were present in almost half (three out of seven) of the PCs, in 22% of the aPTs, and in 11% of the PC patients. In all patients with elevated PTH, calcium levels were normal. In the PA subgroup, all patients had mildly elevated PTH levels (<100 pg/mL), and three out of seven had low vitamin D levels, while another three were on vitamin D treatment for a severe preoperative hypovitaminosis. Subsequent follow-ups carried out by the endocrinologists revealed a PTH normalization or a decreasing tendency in relation to the normalization of vitamin D levels. One of the two patients in the aPT subgroup and one of the three patients in the PC subgroup had similar follow-up profiles. Of the two patients with low PTH at the first follow-up (both in the PA subgroup), only one was diagnosed with persistent hypoparathyroidism at 6 months after surgery (1.2% of the entire cohort of patients).

In all PC cases, and in most (77.78%) aPT cases, follow-ups were performed every 6 months. The mean follow-up time for PC patients was 18 ± 4.9 months (range 14–46), and for aPT patients it was 19.9 ± 10.4 months (range 16–65). There was no significant difference between the aPT and PC subgroups regarding the persistence of disease, and no recurrences were recorded, but the longest follow-up period in our cohort was approximately 4 years. Two PC patients had persistent disease at 14 and 16 months after surgery, respectively. The first one was diagnosed postoperatively with pulmonary metastasis, and the second had a severe preoperative clinical and biochemical profile (hypercalcemic crisis, brown tumors, very low 25-OH vitamin D level). Of the aPT subgroup, one patient had persistent disease at 65 months after surgery, also with progression (rising levels of both calcium and PTH), but without imagistic evidence of disease. This patient also presented a severe preoperative clinical, biochemical, and imagistic profile, more in line with that of PC patients. Interestingly, in both of the aforementioned patients, upon immunohistochemistry, the parafibromin stain was negative and galectin-3 was positive, which might indicate more aggressive tumors with possible recurrences.

In their review of the literature, Saxe et al. found that, in studies with more than 50 nerves at risk during thyroid and parathyroid surgery, the incidence of total (permanent plus transient) and permanent RLN injuries varied between 1.4 and 19.5%, and between 0 and 6.7%, respectively. Also, their meta-analysis of 60 studies that employed IONM during surgery demonstrated a statistically significant effect favoring the use of IONM in reducing the incidence of permanent and total (transient and permanent) RLN injuries [35]. There are only a few dedicated studies regarding the use of IONM in parathyroid surgery [103,104]. Astl et al. reported a postoperative RLN palsy of 0.7% [103]. Mu et al., in their analysis of 270 PHPT cases operated upon with and without IONM, reported permanent RLN injuries in 0% and 0.92%, respectively [104]. In our study, transient and permanent vocal cord palsy was present in 4.82% and 1.20% of all of the patients, respectively, regardless of IONM use. When we compared cases with and without IONM use, the incidence of permanent vocal cord palsy was 0% vs. 2.04% (one case with concomitant extensive thyroid surgery for malignancy). The higher rates of both transient and permanent RLN palsy in our cohort compared to previous studies could be explained by the fact that these results reflect all of the cases, with and without concomitant thyroid surgery.

Some authors have suggested that IONM is useful in high-risk bilateral neck exploration, especially in patients with associated thyrotoxicosis, in retrosternal parathyroid lesions, in suspected malignancy, and in re-interventions [105]. When analyzing only the cases with concomitant thyroid resections, we observed a worrisome incidence of combined transient and permanent vocal cord palsy in both cohorts, with and without IONM use (6.25% and 14.29%, respectively). This could be explained by the associated thyroid pathology (Hashimoto’s thyroiditis in two of the three cases, and the one malignant thyroid case requiring more extensive dissection). Still, neuromonitoring of the RLN did not significantly reduce the incidence of vocal cord palsy in the entire cohort analyzed, even when only cases with concomitant thyroid surgery were evaluated.

Rates of EBSLN injury, although under-reported in the literature, range between 0 and 58% [106,107,108,109]. Several prospective randomized controlled studies have shown that IONM improves the identification rate of the EBSLN and reduces the proportion of patients with subjective voice changes [107,109,110]. In our cohort of parathyroid cases, the incidence of possible EBSLN injury was 15.66%. When we analyzed subtle voice changes possibly related to EBSLN injuries, a non-significant trend toward fewer changes in the monitored group (8.82% vs. 20.41%, *p* = 0.153) was observed. Interestingly, when considering only cases with concomitant parathyroid and thyroid surgery, neuromonitoring was associated with a lower incidence of EBSLN-related voice changes (12.5% vs. 57.14%, *p* = 0.019). To conclude on this matter, in parathyroid surgery, when concomitant thyroid resections are planned (concomitant thyroid pathology) or predicted (preoperative PC suspicion), consideration should be given to IONM use, especially to lower the incidence of EBSLN injuries.

### 4.1. Limitations and Strengths of This Article

This study has several limitations that should be acknowledged. First, although the data were collected prospectively, the retrospective nature of the analysis introduces potential biases, particularly in data completeness. Second, the relatively small sample size, especially for parathyroid carcinoma (PC) and atypical parathyroid tumors (aPTs), may limit the statistical power and generalizability of our findings. Another limitation is the lack of long-term follow-up data, which prevents us from assessing recurrence rates and long-term functional outcomes. Also, the postoperative evaluation of the EBSLN function was based on an empirically proven method. Lastly, as this study was conducted in a single center, our institutional practices and patient demographics may not fully represent broader populations, necessitating multicenter validation studies to confirm our results.

This study contributes valuable insights into rare parathyroid neoplasms, especially aPTs and PC, which are underrepresented in the literature, emphasizing the importance of integrating thorough clinical, biochemical, and imaging analysis, along with intraoperative judgment for accurate diagnosis and optimal surgical management.

### 4.2. Clinical Implications

The findings of this study have several important clinical implications for the diagnosis and surgical management of parathyroid tumors. Firstly, the significant differences in biochemical markers, particularly extreme elevations in PTH and serum calcium, underscore the necessity of considering parathyroid carcinoma (PC) in cases with severe hyperparathyroidism and hypercalcemia. Secondly, the distinct ultrasonographic features associated with malignancy—larger tumor size (≥3 cm), irregular margins, inhomogeneous structure, and increased depth-to-width ratio—highlight the role of preoperative imaging in raising suspicion for PC and guiding surgical planning. Thirdly, intraoperative findings such as firm, adherent, lobulated tumors should prompt heightened but also critical suspicion for malignancy, reinforcing the need for radical *en bloc* resection when appropriate, in order to reduce the risk of recurrence. Additionally, while neuromonitoring did not significantly impact vocal cord paralysis rates, its potential benefit in reducing voice alterations related to the external branch of the superior laryngeal nerve (EBSLN) suggests its selective use, particularly in cases where concomitant thyroid surgery is planned. Integrating these biochemical, imaging, intraoperative, histopathological, and IHC findings into standardized diagnostic and surgical protocols can improve PC suspicion rates, consequent surgical outcomes, and long-term prognosis.

## 5. Conclusions

Our findings confirm that extreme elevations in PTH and serum calcium, along with distinct ultrasonographic features (tumor size ≥ 3 cm, irregular margins, and increased depth-to-width ratio), are strong indicators of malignancy.

Intraoperative assessment further supports the diagnosis, with firm, adherent tumors being more frequently associated with PC. Additionally, intraoperative neuromonitoring showed a potential benefit in reducing voice alterations related to the external branch of the superior laryngeal nerve (EBSLN), particularly in patients undergoing concomitant thyroid surgery.

These findings emphasize the need for a multimodal diagnostic approach, integrating clinical, biochemical, imaging, and intraoperative data to improve early PC suspicion, to optimize surgical treatment and, thus, to favorably influence the outcome. Utilizing all resources available, including IOPTH, laryngeal nerve neuromonitoring, and immunohistochemistry staining, can add extra benefit to the management of these challenging cases. Future multicenter studies with long-term follow-up are warranted to complete these findings and further refine the clinical guidelines for the management of parathyroid tumors.

## Figures and Tables

**Table 1:** Epidemiological, clinical, and biochemical characteristics of the study patients (n = 83).

Diagnosis	PA (n = 67)	aPT (n = 9)	PC (n = 7)	*p*-Value
Age, median (IQR)	54 (45.5–65)	64 (56–68)	57 (51–69)	0.199
Gender:				0.156
Female, n (%)	57 (85.07)	8 (88.89)	4 (57.14)	
Male, n (%)	10 (14.93)	1 (11.11)	3 (42.86)	
Palpable tumor, n (%)	0 (0)	1 (11.11)	1 (14.29)	0.035
Hypercalcemic crisis, n (%)	0 (0)	1 (11.11)	1 (14.29)	0.035
Combined bone + kidney involvement	10 (14.93)	4 (44.44)	6 (85.71)	<0.001
Other systems affected by disease:				<0.001
0, n (%)	32 (47.76)	2 (22.22)	1 (14.29)	
1, n (%)	17 (25.37)	1 (11.11)	0 (0)	
2, n (%)	15 (22.39)	2 (22.22)	1 (14.29)	
>2 systems, n (%)	3 (4.48)	4 (44.44)	5 (71.43)	<0.001
PTH (pg/mL), median (IQR)	168.05 (127.58–229.75)	336.5 (189–1210)	788.6 (421.72–1310.17)	<0.001
PTH ≥ 5xNV, n (%)	5 (7.46)	5 (55.56)	6 (85.71)	<0.001
Albumin-corrected serum calcium (mg/dL), median (IQR)	11.01 (10.45–11.8)	11.37 (11.19–13.7)	14.12 (12.65–14.6)	0.006
Albumin-corrected serum calcium ≥14 mg/dL, n (%)	1 (1.49)	3 (33.33)	5 (71.43)	<0.001
Serum 25-OH vitamin D (ng/mL), median (IQR)	24.85 (21.54–32.91)	26.56 (23.94–29.61)	29.41 (23.32–30.34)	0.975
Serum 25-OH vitamin D < NV, n/n′ (%)	19/32 (59.38)	4/7 (57.14)	4/7 (57.14)	1
Anti-TPO/anti-TG Ab > NV, n/n′ (%)	8/32 (25)	1/5 (20)	2/4 (50)	0.587

Ab—antibody (anti-TPO antithyroidperoxidase, anti-Tg antithyroglobulin); aPT—atypical parathyroid tumor; IQR—interquartile range; n—number of identified cases; n′—number of evaluated cases; NV—normal value; PA—parathyroid adenoma; PC—parathyroid carcinoma.

**Table 2:** Ultrasound features in the different study subgroups.

Diagnosis	PA (n = 67)	aPT (n = 9)	PC (n = 7)	*p*-Value
Patients without parathyroid identification on US, n (%)	10 (14.93)	1 (11.11)	0 (0)	-
US: maximum parathyroid diameter (cm), median (IQR)	1.8 (1.3–2.5)	3.05 (2.5–3.28)	3.4 (2.75–5)	<0.001
US: parathyroid largest diameter ≥3 cm, n/n′ (%)	2/57 (3.51)	4/8 (50)	5/7 (71.43)	<0.001
US: parathyroid D/W, median (IQR)	0.67 (0.5–0.82)	0.65 (0.57–0.75)	1.07 (1.05–1.1)	0.005
US: parathyroid D/W ≥ 1, n/n′ (%)	10/57 (17.54)	2/8 (25)	6/7 (85.71)	0.003
US: suspicious parathyroid features (irregular shape, obscure or infiltrative borders, thick capsule), n/n′ (%)	2/57 (3.51)	3/8 (37.5)	6/7 (85.71)	<0.001
US: suspicious parathyroid features (heterogeneous texture, calcifications, cystic changes), n/n′ (%)	5/57 (8.77)	4/8 (50)	6/7 (85.71)	<0.001

aPT—atypical parathyroid tumor; D/W—depth/width ratio; IQR—interquartile range; n—number of identified cases; n′—number of evaluated cases; PA—parathyroid adenoma; PC—parathyroid carcinoma; US—ultrasound.

**Table 3:** Surgical data.

Diagnosis:	PA (n = 67)	aPT (n = 9)	PC (n = 7)	*p*-Value
Cervicotomy with BPE, n (%)	64 (95.52)	9 (100)	7 (100)	-
MIVAP with UPE, n (%)	3 (4.48)	0 (0)	0 (0)	-
Suspicious parathyroid features: adherence to the thyroid, n (%)	5 (7.546)	1 (11.11)	5 (71.43)	<0.001
Thyroid capsule adherences to surrounding tissues, n (%)	6 (8.96)	1 (11.11)	0 (0)	0.776
Suspicious parathyroid features: grayish-white, lobulated, firm, n (%)	3 (4.48)	4 (44.44)	6 (85.71)	<0.001
PE only, n (%)	47 (70.15)	5 (55.56)	1 (14.29)	0.033
*En* bloc resection, n (%)	10 (14.93)	2 (22.22)	6 (85.71)	<0.001
Concomitant thyroid surgery including *en bloc* resection, n (%)	20 (29.85)	4 (44.44)	6 (85.71)	0.009
sIOPTH, n/n′ (%)	57/67 (85.07)	6/9 (66.67)	7/7 (100)	-
>50% drop, n (%) confusing data	56 (98.24) 1 (1.75)	6 (100) 0 (0)	7 (100) 0 (0)	1
Neuromonitoring, n (%)	28 (41.79)	3 (33.33)	3 (42.86)	0.918

aPT—atypical parathyroid tumor; BPE—bilateral parathyroid exploration; MIVAP—minimally invasive video-assisted parathyroidectomy; n—number of identified cases, n′—number of evaluated cases; PA—parathyroid adenoma; PE—parathyroid excision; PC—parathyroid carcinoma; sIOPTH—surrogate intraoperative parathormone assay; UPE—unilateral parathyroid exploration.

**Table 4:** *En bloc* resections in PC, aPT, and PA—comparative data regarding pre- and intraoperative suspicions and associated thyroid pathology.

Dg-n	Clinical Suspicion	Biochemical Suspicion		Ultrasound Suspicion			Intraoperative Suspicion		Thyroid Pathology with Surgical Indication
	>2 syst. Affected	Ca ≥ 14 mg/dL	PTH > 5xNV	App.	D/W ≥ 1	Parathyroid > 3 cm	Adhesions	App.	
PC-1	+	-	+	+	+	+	+	+	-
PC-2	+	+	+	+	+	+	-	+	-
PC-3	+	+	+	+	+	+	+	+	-
PC-4	-	-	+	+	+	-	+	+	PTCs
PC-5	+	+	+	+	+	+	+	+	-
PC-6	+	+	+	+	+	+	+	+	PTCs
aPT-1	+	+	+	-	+	+	-	-	N-G
aPT-2	-	-	-	+	-	+	-	+	N-G
PA-1	-	-	-	-	+	-	+	-	N-G + H-T
PA-2	-	-	+	+	-	+	-	+	N-G
PA-3	-	-	-	-	-	-	-	-	N-G
PA-4	-	-	-	-	-	-	-	-	N-G
PA-5	-	-	-	-	+	-	-	-	N-G
PA-6	-	-	-	-	-	-	-	-	N-G
PA-7	-	-	-	-	-	-	-	-	PTCs + H-T
PA-8	-	-	-	UI	UI	UI	-	-	PTCs
PA-9	-	-	-	-	-	-	+	-	N-G + H-T
PA-10	-	-	-	+	-	-	+	-	N-G + H-T

aPT—atypical parathyroid tumor; App.—appearance (US or macroscopic); Dg-n—diagnosis-number; D/W—depth/width ratio; H-T –Hashimoto’s thyroiditis; N-G—nodular goiter; NV—normal value; PC—parathyroid carcinoma; PA—parathyroid adenoma; PTC—papillary thyroid carcinoma; syst.—systems; UI—unidentified on US; “+”—presence; “-”—absence.

**Table 5:** Immunohistochemistry findings in aPT and PC.

Diagnosis	PA (n = 67)	aPT (n = 9)	PC (n = 7)	*p*-Value
Ki-67 (%), median (IQR)	1 (0.5–2)	2 (1–3)	5 (2.3–5)	0.008
Ki-67 > 5%, n (%)	3 (5)	0 (0)	4 (57.14)	0.001
Parafibromin stain, n. (%)				0.035
Negative	0 (N/A)	1 (11.11)	2 (28.57)	
Weak positive	0 (N/A)	0 (0)	3 (42.86)	
Positive	0 (N/A)	8 (88.89)	2 (28.57)	
Galectin-3 (positive stain), No. (%)	0 (N/A)	1 (11.11)	2 (28.57)	0.629

aPT—atypical parathyroid tumor; IQR—interquartile range; PA—parathyroid adenoma; PC—parathyroid carcinoma.

**Table 6:** Concomitant thyroid pathology.

Diagnosis	PA (n = 67)	aPT (n = 9)	PC (n = 7)	*p*-Value
Associated thyroid disease: hyper- or hypofunction, Hashimoto’s thyroiditis, large diffuse goiter, n (%)	20 (29.85)	2 (22.22)	3 (42.86)	0.744
Nodular thyroid disease	35 (52.24)	4 (44.44)	3 (42.86)	0.963
Cancer suspicion on US, n (%)				0.655
Yes	7 (10.61)	0 (0)	2 (28.57)	
No	28 (42.42)	4 (44.44)	1 (14.29)	
Without nodules	32 (47.76)	5 (55.56)	4 (57.14)	
Histopathology of resected thyroid, n (%):				0.018
Malignant	7 (10.45)	0 (0)	2 (28.57)	
Benign	13 (19.40)	4 (44.44)	4 (57.14)	
Without thyroid operation	47 (70.15)	5 (55.56)	1 (14.29)	

aPT—atypical parathyroid tumor; PA—parathyroid adenoma; PC—parathyroid carcinoma; US—ultrasound.

**Table 7:** Follow-up data at 45 days after surgery.

Diagnosis	PA (n = 67)	aPT (n = 9)	PC (n = 7)	*p*-Value
PTH level, n (%) Normal Low High	58 (86.57) 2 (2.99) 7 (10.45)	7 (77.78) 0 (0) 2 (22.22)	4 (57.14) 0 (0) 3 (42.86)	0.128
Low 25-OH vitamin D, n (%)	31 (51.67)	5 (55.56)	3 (42.86)	0.922
RLN—vocal cord palsy, n (%)	3 (4.48)	1 (11.11)	1 (14.29)	0.245
EBSLN-related voice changes (low amplitude, fatigue, “S” and “Z” < 5 s), n (%)	7 * (10.45)	3 * (33.33)	3 * (42.86)	0.024

aPT—atypical parathyroid tumor; EBSLN—external branch of the superior laryngeal nerve; PA—parathyroid adenoma; PC—parathyroid carcinoma; RLN—recurrent laryngeal nerve. * Patients with vocal cord palsy or hypomobility were excluded.

**Table 8:** Laryngeal nerve neuromonitoring and outcomes.

Laryngeal Nerve Neuromonitoring	Yes	No	*p*-Value
All cases, n (%)	34 (40.96)	49 (59.04)	-
RLN—vocal cord palsy, n (%)	2 (5.88)	3 (6.12)	1
Transient	2	2	
Permanent	0	1	
EBSLN-related voice changes (low amplitude, fatigue, “S” and “Z” < 5 s), n (%)	3 (8.82)	10 (20.41)	0.153
Transient	2	3	
Permanent	1	7	
Only cases with concomitant thyroidectomy, n (%)	16 (53.33)	14 (46.67)	-
RLN—vocal cord palsy, n (%)	1 (6.25)	2 (14.29)	0.586
Transient	1	1	
Permanent	0	1	
EBSLN-related voice changes (low amplitude, fatigue, “S” and “Z” < 5 s), n (%)	2 (12.5)	8 (57.14)	0.019
Transient	1	2	
Permanent	1	6	

EBSLN—external branch of the superior laryngeal nerve; RLN—recurrent laryngeal nerve.

## Data Availability

The data presented in this study are available upon request from the corresponding authors.
